# Chinese parents’ intention to vaccinate their 0–5-year-old children with the EV-71 vaccine against hand, foot, and mouth disease and willingness-to-pay

**DOI:** 10.3389/fpubh.2024.1336687

**Published:** 2024-03-07

**Authors:** Lu Cheng, Sumei Zhong, Xiaonan Xu, Junrong Li, Fangqin Xie, Yulan Lin, Dongjuan Zhang

**Affiliations:** ^1^Department of Epidemiology and Health Statistics, School of Public Health, Fujian Medical University, Fuzhou, Fujian, China; ^2^Vaccine Clinical Trial Center, Fujian Provincial Center for Disease Control and Prevention, Fuzhou, Fujian, China

**Keywords:** HFMD, EV-71 vaccine, willingness-to-pay, children, China

## Abstract

**Background:**

This study aimed to determine the intention and willingness-to-pay (WTP) of Chinese parents/guardians to vaccinate their children with the EV-71 vaccine. Knowledge levels about hand, foot, and mouth disease (HFMD) and the EV-71 vaccine were also investigated.

**Methods:**

A cross-sectional, self-administered online survey was conducted between November 2022 and March 2023. A stratified multi-stage random sampling method was used to recruit parents/guardians of children aged 0–5 years in southeastern China.

**Results:**

A total of 3,626 complete responses were received. The mean knowledge score of HFMD was 9.99 (±4.23) out of a total of 14 points. The majority of the participants reported a somewhat willing intent (58.8%), followed by an extremely willing intent (28.9%). Participants who did not consider the EV-71 vaccine expensive (OR = 2.94, 95%CI 2.45–3.53) perceived that the EV-71 vaccine is effective (OR = 2.73, 95%CI 1.52–4.90), and a high knowledge level of HFMD (OR = 1.90, 95%CI 1.57–2.29) had the highest significant odds of having an extremely willing intent to vaccinate their children with the EV-71 vaccine. The median (interquartile range [IQR]) of WTP for the EV-71 vaccine was CNY¥200/USD$28 (IQR CNY¥100-400/USD$14-56). The highest marginal WTP for the vaccine was mainly influenced by the perceived high cost of the vaccine. Those participants who did not consider the EV-71 vaccine expensive had more than 10 times higher odds of vaccinating their children (OR = 10.86, 95%CI 8.49–13.88). Perceived susceptibility, perceived benefits, and perceived barriers were also significant influencing factors in the highest marginal WTP.

**Conclusion:**

The findings demonstrate the importance of improving health promotion and reducing the barriers to EV-71 vaccination. Therefore, it is important to improve health promotion and reduce the barriers to EV-71 vaccination.

## 1 Introduction

Hand, foot, and mouth disease (HFMD) is a global enteroviral infection that mainly affects children aged 0–5 years. The main clinical manifestations are fever, vesicular rashes on the hands, feet, and buttocks, and ulcers in the oral mucosa. Usually, HFMD is self-limiting, but a small proportion of children may experience severe complications such as meningitis, encephalitis, acute flaccid paralysis, and neurorespiratory syndrome (1–3).

HFMD can be caused by a variety of enteroviruses, among which enterovirus 71 (EV-71) is the leading pathogen and could cause severe illness and death (4–6). Between 1970 and 2000, a large number of outbreaks of HFMD associated with EV-71 occurred in the Asia-Pacific region (7). The severe neurological symptoms of HFMD caused by EV-71 have made HFMD a serious public health problem for children in Asia–Pacific countries, with China being one of the most severely affected countries (8, 9). During 2008 and 2017, a total of 13.8 million cases of HFMD were reported in China, including 3,300 deaths, resulting in a fatality rate of approximately 0.2 per 1,000 deaths (10). EV-71-associated HFMD placed a significant burden on infected children, their families, and society, and the highest mortality rate of EV-71-associated HFMD also occurred in China (1.8%) (11, 12). Unfortunately, there are no specific therapeutic drugs for HFMD, and the use of vaccines is the most effective measure to prevent this disease (13). China’s self-developed inactivated EV-71 vaccine has been marketed since its first approval in December 2015 (14). However, the vaccination rate remains low across China (15, 16). During 2016 and 2019, the coverage rates for doses 1 and 2 were 24.7 and 19.4%, respectively, in Guangzhou City (16). Similarly, the whole-course vaccination rates of two doses of the EV-71 vaccine in Fujian Province were 4.2 and 8.5% in 2017 and 2018, respectively (17). Parental acceptance of the EV-71 vaccination is crucial to enable public health systems to reach the recommended threshold and achieve herd immunity, thereby halting the virus spread.

Parental decision-making for the vaccination of their children is complex and impacted by many factors, depending on parents’ perspectives. The health belief model (HBM) explains and predicts health behaviors by focusing on the attitudes and beliefs of individuals (18). This model includes concepts such as perceived susceptibility, perceived severity, perceived benefits, perceived barriers, cues to action, and self-efficacy. The HBM has been extensively used to study vaccination beliefs and behaviors to identify parental behavioral intentions toward childhood vaccination (19, 20). HBM constructs have been recognized as an important predictor of influenza vaccine uptake in many previous influenza vaccination studies (21, 22), including a study on Chinese parents’ decisions to vaccinate their children against seasonal influenza (23).

In China, the EV-71 vaccine is a class II vaccine and requires out-of-pocket payments. Therefore, determining parents’ willingness-to-pay (WTP) for the EV-71 vaccine is critical for future pricing and for increasing vaccination rates and coverage. In monetary terms, WTP refers to the maximum amount that an individual would be willing to sacrifice to obtain the benefits of a program, service, or health technology (24). In vaccination decisions, the decision to vaccinate depends on the WTP of an individual for a vaccination to obtain increased health benefits (25). HBM constructs have also been used to explain WTP for influenza vaccination (26).

More evidence about parental acceptance and the WTP for the EV-71 vaccine is essential to promote the implementation of the vaccination program in China and also to provide insights into future pricing considerations, demand forecasts, and the implementation of the national EV-71 immunization program. Therefore, the main objective of this study was to determine Chinese parental intention to vaccinate their children aged 0–5 years with the EV-71 vaccine against HFMD. The HBM was used as the framework to predict parental intention to vaccinate their children and the highest marginal WTP.

## 2 Materials and methods

### 2.1 Study participants and study design

We commenced a cross-sectional, web-based anonymous survey between November 2022 and March 2023 in Fujian Province. Study participants were parents or guardians (e.g., grandparents) of children aged 0–5 years who have not received the EV-71 vaccine. Participants need to be long-term residents of Fujian Province, i.e., they should have lived in the study area for at least 6 months in the 12 months before the survey.

A stratified multi-stage random sampling method was adopted to select study regions. First, urban and rural areas in all 10 cities (36 urban areas and 53 rural areas) of Fujian Province were ranked by population size. Systematic sampling was then used to select 20 urban regions and 16 rural regions. Second, one vaccination center (community health center) was selected using the systematic sampling method from each selected region as the study center. Third, each study center recruited at least 100 parents/guardians of children aged 0–5 years to complete the online survey. One medical staff from each selected vaccination center was recruited and trained in a standard manner to recruit study participants and provide an explanation of the questionnaire. The participants were informed that their participation was voluntary and that consent was implied through their completion of the questionnaire.

### 2.2 Instruments

The survey consisted of questions that assessed (1) demographic background, health status, and history of HFMD; (2) knowledge about HFMD and the EV-71 vaccine; (3) information source of the EV-71 vaccine; (4) perception of HFMD and EV-71 vaccination based on HBM; and (5) intention and WTP for the EV-71 vaccine. In cases where a family has more than one child aged 0–5 years, participants were requested to provide data based on the second child.

#### 2.2.1 Demographics, health status, and history of HFMD

Personal details, including age, gender, place of birth, workplace, census register place, highest education level, number of children aged 0–5 years, occupation, and average monthly household income, were collected. The participants were also asked whether their children had a history of HFMD and to rate their overall health status.

#### 2.2.2 Knowledge about HFMD and the EV-71 vaccine

The participants’ knowledge was assessed using a series of questions regarding signs and symptoms of HFMD infection, transmission, susceptible population, viruses, and the EV-71 vaccine (a 14-item scale). The response options were “true,” “false,” or “do not know.” A correct response was given a score of one, and an incorrect response or “do not know” response was scored zero. The possible total knowledge score ranged from 0 to 14, with higher scores representing higher levels of knowledge. The reliability of HFMD knowledge items was evaluated by assessing the internal consistency of the items representing the knowledge score. The 14 knowledge items had a reliability (Cronbach’s alpha) of 0.915.

#### 2.2.3 Information source for the EV-71 vaccine

Participants were asked to provide their information regarding the EV-71 vaccine from the following options: medical institutions, family/friends, social media, TV/radio/newspapers/magazines, Internet, school, community health promotion, and children’s feedback.

#### 2.2.4 Perception of HFMD and EV-71 vaccination

HBM-derived items were used to measure the participants’ perceptions of HFMD and the EV-71 vaccination (27, 28). The questions probed perceived susceptibility to HFMD (two items), perceived severity of HFMD (two items), perceived benefits of an EV-71 vaccine (two items), perceived barriers to getting a vaccination against HFMD (four items), and cues to action (two items). All the response options were either ‘Yes’ or ‘No.’

#### 2.2.5 Intention to receive an EV-71 vaccine and willingness-to-pay (WTP)

The intention to accept an EV-71 vaccine was measured using a one-item question (“Do you want to vaccinate your child with EV-71 vaccine?”) on a 4-point scale (“extremely unwilling,” “somewhat unwilling,” “somewhat willing,” and “extremely willing”). WTP was measured using a one-item question (“What is the maximum amount you are willing to pay for the EV-71 vaccine?”) on a 9-point scale (CNY¥ 100/USD$ 14 to CNY¥ 900/USD$ 126, at a currency ratio of 7:1). The price range options were based on the approximate minimum–maximum price range of currently available vaccines in China.

### 2.3 Sample size calculation

The sample size was calculated for each region using the equation: *n* = Z^2^ P(1-P)/d^2^ (29), in which the prevalence rate was 70% (P) based on the vaccine acceptance rate reported in the previous literature (30, 31). The significance level was set at 0.05, and the allowable error was 0.03 (d). The minimal sample size required for this study was 896.

### 2.4 Statistical analysis

We conducted both univariate and multivariable logistic regression analyses, including all factors showing significance (*p* < 0.05). Odds ratios (OR), 95% confidence intervals (95%CI), and *p*-values were calculated for each independent variable. The model fit of the multivariable logistic regression analysis was assessed using the Hosmer–Lemeshow goodness-of-fit test (32). The majority of respondents were willing to pay between CNY¥100 and CNY¥400 for the EV-71 vaccine, and a lower proportion reported a WTP of CNY¥ 500 and above. Hence, the WTP was divided into three price ranges (CNY¥ 100/200, CNY¥ 300/400, and CNY¥ 500 and above). A multivariable multinomial logistic regression was employed to model factors associated with marginal WTP for the EV-71 vaccine for three price ranges, with CNY¥ 100/200, the lowest coded category, as the reference group. Similarly, only significant factors in the univariate analyses (*p* < 0.05) were selected for the multinomial logistic regression analysis. A *p*-value of less than 0.05 was considered statistically significant. All statistical analyses were performed using the Statistical Package for the Social Sciences version 20.0 (IBM Corp., Armonk, NY, USA).

## 3 Results

### 3.1 Demographics

A total of 3,626 complete responses were received (Table 1). The age range of participants was 16–76 years, with a mean age of 33.55 years (standard deviation, SD ± 7.73). The majority of participants were aged 30 to 40 years (58.8%), were mothers (70.7%), were from Fujian households (92.7%), were working in urban areas (61.7%), and had a family monthly income of < CNY¥8,000 (69.1%). Most of the families have only one child (80.4%), with almost equal proportions of boys (56.7%) and girls (43.3%). Only 8.7% of children have had HFMD before.

**Table 1:** Demographic characteristics of participants (*n* = 3,626).

Characteristics	n (%)
Guardian factor	
Age (years)	
<30	1,049 (28.9)
30–40	2,133 (58.8)
>40	444 (12.3)
Relationship	
Father	875 (24.1)
Mother	2,563 (70.7)
Other guardians	188 (5.2)
Place of birth	
Urban	1,208 (33.3)
Rural	2,418 (66.7)
Workplace	
Urban	2,236 (61.7)
Rural	1,390 (38.3)
Census register place	
Fujian Province	3,361 (92.7)
Other provinces	265 (7.3)
Highest education level	
Primary school and below	185 (5.1)
Secondary school	886 (24.5)
High school	774 (21.3)
University and above	1,781 (49.1)
Number of children aged 0–5 years	
1	2,916 (80.4)
>1	710 (19.6)
Occupation category	
Professionals and managerial	911 (25.1)
Clerks	237 (6.5)
Industrial workers	318 (8.8)
Self-employed	411 (11.3)
Farmers	431 (11.9)
Service personnel	164 (4.5)
Housewife/retiree/unemployed	663 (18.3)
Others occupation	491 (13.5)
Average annual household income (CNY¥)	
<4,000	1,071 (29.5)
4,000–7,999	1,436 (39.6)
8,000–15,999	953 (26.3)
≥16,000	166 (4.6)
Knowledge score of HFMD	
High (12–14)	1,765 (48.7)
Low (0–11)	1,861 (51.3)
Mean (std)	9.99 (±4.23)
Children factor	
Child’s gender	
Boy	2,057 (56.7)
Girl	1,569 (43.3)
Child’s age (years)	
<3	2,089 (57.6)
3–5	1,537 (42.4)
Child’s health status	
Healthy	3,348 (92.3)
Frequently sick	278 (7.7)
Child’s category	
Nursery children	1,741 (48.0)
Non-nursery children	1,885 (52.0)
Child’s HFMD history	
Yes	314 (8.7)
No	3,312 (91.3)

### 3.2 Knowledge about HFMD and the EV-71 vaccine

**Figure 1:**
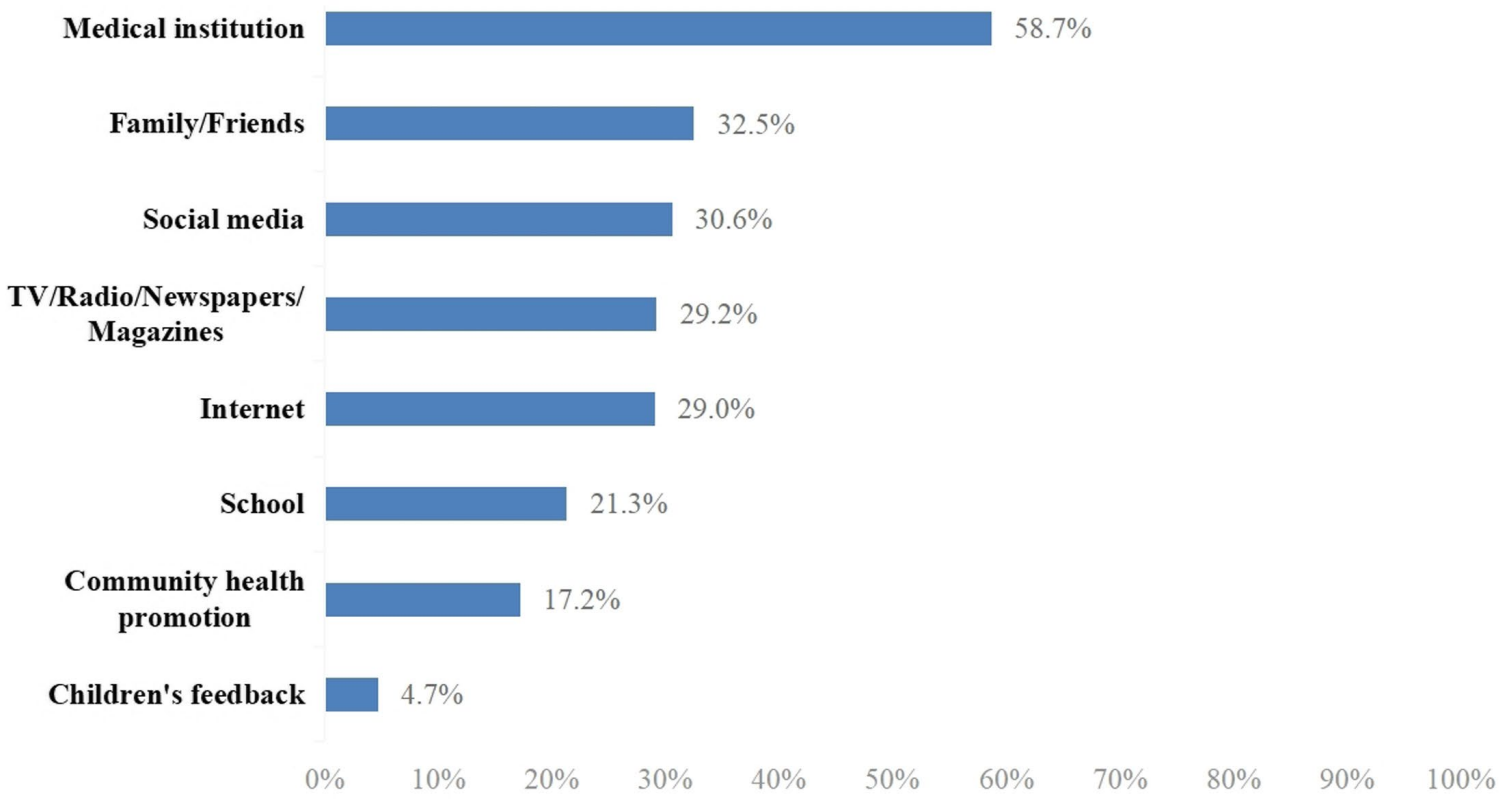
EV-71 vaccination information sources (*N* = 2,448).

The mean of the total knowledge score was 9.99 (SD ± 4.23) out of a possible score of 14. The median was 11 (interquartile range, IQR, 8–14). The knowledge scores were categorized as a score of 0–11 or 12–14 based on the median split; as such, a total of 1,765 (48.7%) participants were categorized as having a score of 12–14 and 1,861 (51.3%) participants had a score of 0–11. The majority (92.2%) of participants had heard of HFMD, but only 52.5% of them were aware of the EV-71 disease and 67.5% were aware of the EV-71 vaccine (data not shown). Figure 1 shows that the major information source was a medical institution (58.7%), followed by family/friends (32.5%), social media (30.6%), TV/radio/newspapers/magazine (29.2%), Internet (29.0%), school (21.3%), community health promotion (17.2%), and children’s feedback (4.7%).

### 3.3 Health beliefs

The participants had high perceptions of susceptibility. Nearly half of the participants agreed that their children had a high risk of getting HFMD (45.4%), and the majority were worried about the likelihood of their children getting HFMD (82.7%). The participants also had high perceptions of the severity of HFMD; 50.8% agreed that a child could became very sick if infected with HFMD, and 89.9% agreed that HFMD infection could affect a child’s schooling. High perceptions of the benefits of the EV-71 vaccination were reported. The majority (91.4%) of the participants perceived the benefit of the EV-71 vaccination in preventing HFMD. Under the perceived barriers construct, concerns about the side effects, affordability, and safety were reported by 54.7, 54.1, and 53.0%, respectively. Receiving information from medical staff (74.2%) was the major construct of cues to action.

### 3.4 EV-71 vaccination intent

**Figure 2:**
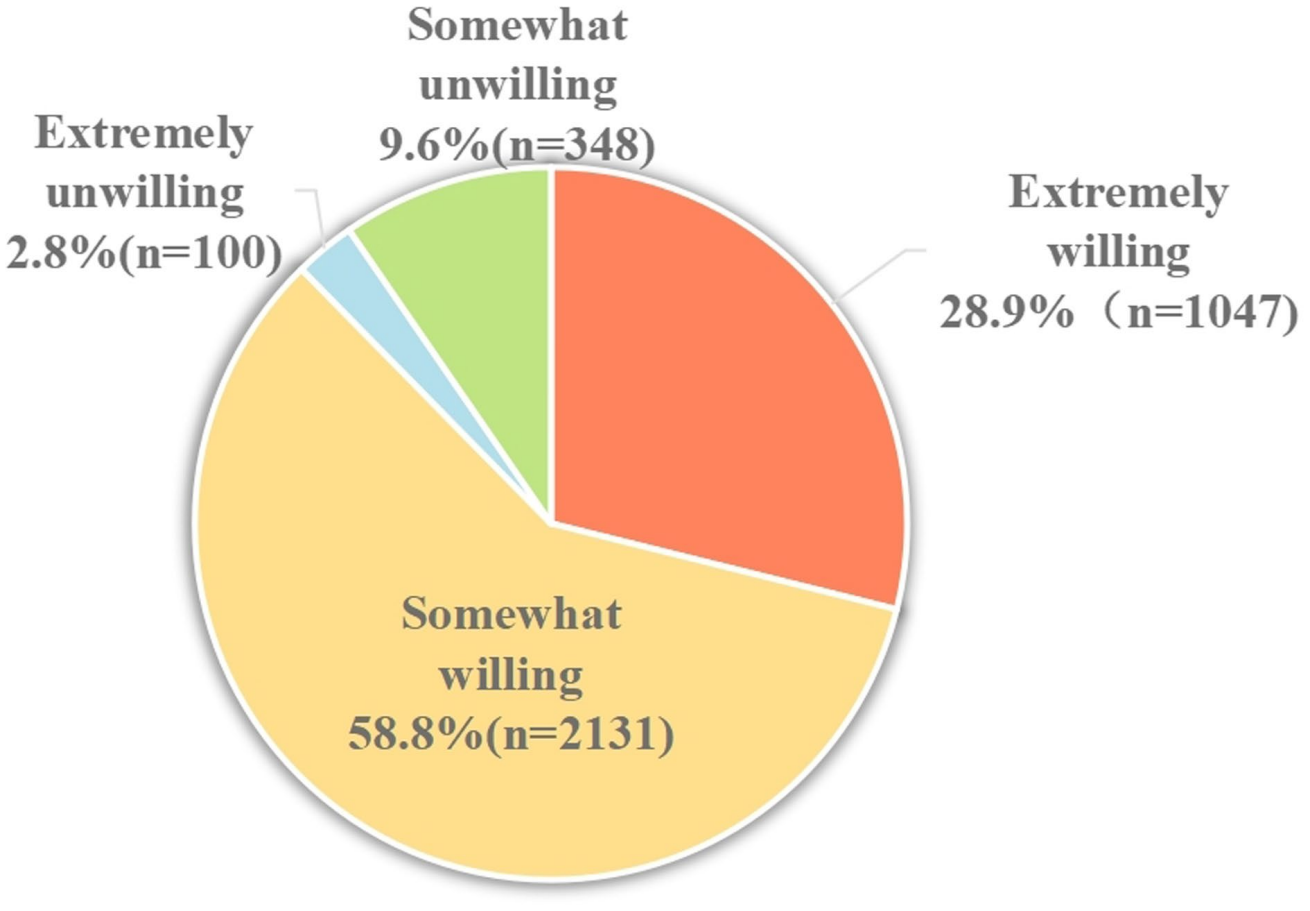
EV-71 vaccination intent (*N* = 3,626).

Figure 2 shows the proportion of responses with the intention to vaccinate their children with the EV-71 vaccine. A total of 3,178 (87.7%) participants responded “Yes” with the intention to vaccinate their children with EV-71 vaccine, while only 448 (12.4%) responded “No.” In a more specific breakdown, the majority responded somewhat willing (58.8%), followed by extremely willing (28.9%). Only 2.8% responded as extremely unwilling, and 9.6% reported as somewhat unwilling. The third and fourth columns of Table 2 show the responses of extremely willing against the other options (somewhat willing/somewhat unwilling/extremely unwilling) to the intention to vaccinate by demographics and health belief constructs. By demographics, multivariable analyses revealed that being self-employed (OR = 1.54, 95% CI: 1.11–2.14), average annual household income ≥CNY¥16,000 (OR = 1.62, 95% CI: 1.07–2.44), and having a child aged less than 3 years (OR = 1.65, 95%CI: 1.33–2.03) were strong significant factors in having a definite intention to vaccinate their children against HFMD. Those with university and above education level (OR = 1.31, 95% CI: 1.07–1.61) and high knowledge score (OR = 1.90, 95% CI: 1.57–2.29) were also significant associated with a definite intention to get their child vaccinated with the EV-71 vaccine.

**Table 2:** Univariate and multivariable analyses of factors associated with willingness to receive the EV-71 vaccine (*N* = 3,626).

		Univariate analysis				Multivariate logistic regression^†^	
	N (%)	Extremely willing (*N* = 1,047)	Somewhat willing/Somewhat unwilling/Extremely unwilling (*N* = 2,579)	OR (95%CI)	value of *p*	Extremely willing vs. Somewhat willing/Somewhat unwilling/Extremely unwilling OR (95%CI)	value of *p*
Social demographic characteristics							
Guardian factor							
Age (years)							
<30	1,049 (28.9)	366 (34.9)	683 (65.1)	2.05 (1.58–2.67)	<0.001	1.11 (0.77–1.60)	0.579
30–40	2,133 (58.8)	589 (27.6)	1,544 (72.4)	1.46 (1.14–1.87)		0.87 (0.62–1.23)	0.435
>40	444 (12.3)	92 (20.7)	352 (79.3)	Reference		Reference	
Relationship							
Father	875 (24.1)	242 (27.7)	633 (72.3)	1.94 (1.28–2.93)	<0.001	1.36 (0.79–2.34)	0.273
Mother	2,563 (70.7)	774 (30.2)	1,789 (69.8)	2.19 (1.48–3.25)		1.57 (0.92–2.68)	0.101
Other guardians	188 (5.2)	31 (16.5)	157 (83.5)	Reference		Reference	
Birthplace							
Urban	1,208 (33.3)	378 (31.3)	830 (68.7)	1.19 (1.02–1.38)	0.023	0.96 (0.79–1.17)	0.670
Rural	2,418 (66.7)	669 (27.7)	1,749 (72.3)	Reference		Reference	
Workplace							
Urban	2,236 (61.7)	692 (3 0.9)	1,544 (69.1)	1.31 (1.12–1.52)	<0.001	0.89 (0.72–1.09)	0.251
Rural	1,390 (38.3)	355 (25.5)	1,035 (74.5)	Reference		Reference	
Census register place							
Fujian Province	3,361 (92.7)	954 (28.4)	2,407 (71.6)	Reference	0.020	Reference	
Other provinces	265 (7.3)	93 (35.1)	172 (64.9)	1.36 (1.05–1.78)		0.79 (0.58–1.09)	0.147
Occupation							
Professionals and managerial	911 (25.1)	313 (34.4)	598 (65.6)	1.61 (1.28–2.01)	<0.001	1.17 (0.87–1.57)	0.290
Clerks	237 (6.5)	86 (36.3)	151 (63.7)	1.75 (1.27–2.40)		1.21 (0.82–1.79)	0.332
Industrial workers	318 (8.8)	69 (21.7)	249 (78.3)	0.85 (0.61–1.17)		0.96 (0.66–1.40)	0.820
Self-employed	411 (11.3)	142 (34.5)	269 (65.5)	1.62 (1.24–2.12)		1.54 (1.11–2.14)	0.010
Farmers	431 (11.9)	80 (18.6)	351 (81.4)	0.70 (0.52–0.94)		0.95 (0.66–1.36)	0.767
Service personnel	164 (4.5)	46 (28.0)	118 (72.0)	1.20 (0.82–1.76)		1.16 (0.75–1.82)	0.505
Others occupation	491 (13.5)	148 (30.1)	343 (69.9)	1.32 (1.02–1.72)		1.24 (0.91–1.69)	0.170
Housewife/Retiree/unemployed	663 (18.3)	163 (24.6)	500 (75.4)	Reference		Reference	
Highest education level							
High school and below	1,845 (50.9)	429 (23.3)	1,416 (76.7)	Reference	<0.001	Reference	
University and above	1,781 (49.1)	618 (34.7)	1,163 (65.3)	1.75 (1.52–2.03)		1.31 (1.07–1.61)	0.020
Average annual household income (CNY¥)							
<4,000	1,071 (29.5)	233 (21.8)	838 (78.2)	Reference	<0.001	Reference	
4,000–7,999	1,436 (39.6)	425 (29.6)	1,011 (70.4)	1.41 (1.01–1.97)		1.26 (0.86–1.85)	0.238
8,000–15,999	953 (26.3)	320 (33.6)	633 (66.4)	1.69 (1.22–2.35)		1.33 (0.91–1.96)	0.146
≥16,000	166 (4.6)	69 (41.6)	97 (58.4)	2.55 (1.82–3.60)		1.62 (1.07–2.44)	0.023
Number of children aged 0–5 years in the family							
1	2,916 (80.4)	844 (28.9)	2,072 (71.1)	1.01 (0.85–1.22)	0.853		
>1	710 (19.6)	203 (28.6)	507 (71.4)	Reference			
Knowledge score of HFMD							
High (12–14)	1,765 (48.7)	686 (38.9)	1,079 (61.1)	2.64 (2.28–3.07)	<0.001	1.90 (1.57–2.29)	
Low (0–11)	1,861 (51.3)	361 (19.4)	1,500 (80.6)	Reference		Reference	<0.001
Children’s factor							
Child’s gender							
Boy	2,057 (56.7)	571 (27.8)	1,486 (72.2)	Reference	0.090		
Girl	1,569 (43.3)	476 (30.3)	1,093 (69.7)	0.88 (0.76–1.02)			
Child’s age (years)							
<3	2,089 (57.6)	728 (34.8)	1,361 (65.2)	2.04 (1.75–2.38)	<0.001	1.65 (1.33–2.03)	<0.001
3–5	1,537 (42.4)	319 (20.8)	1,218 (79.2)	Reference		Reference	
Child’s physical condition							
Healthy	3,348 (92.3)	989 (29.5)	2,359 (70.5)	1.59 (1.18–2.14)	0.002	1.13 (0.81–1.58)	0.480
Frequently sick	278 (7.7)	58 (20.9)	220 (79.1)	Reference		Reference	
Child’s category							
Nursery children	1,741 (48.0)	435 (25.0)	1,306 (75.0)	Reference	<0.001	Reference	
Non-nursery children	1,885 (52.0)	612 (32.5)	1,273 (67.5)	1.44 (1.25–1.67)		0.91 (0.75–1.11)	0.366
Child’s HFMD history							
Yes	314 (8.7)	81 (25.8)	233 (74.2)	Reference	0.208		
No	3,312 (91.3)	966 (29.2)	2,346 (70.8)	1.18 (0.91–1.54)			
Other children around who have suffered from HFMD							
Yes	1,133 (31.2)	339 (29.9)	794 (70.1)	1.08 (0.92–1.26)	0.349		
No/unsure	2,493 (68.8)	708 (28.4)	1,785 (71.6)	Reference			
Health belief							
Perceived susceptibility							
My child has a high chance of getting HFMD							
Yes	1,648 (45.4)	574 (34.8)	1,074 (65.2)	1.70 (1.47–1.97)	<0.001	1.25 (1.02–1.52)	0.030
No	1,978 (54.6)	473 (23.9)	1,505 (76.1)	Reference		Reference	
Worry about my child getting HFMD							
Yes	2,997 (82.7)	955 (31.9)	2,042 (68.1)	2.73 (2.16–3.45)	<0.001	1.79 (1.34–2.37)	<0.001
No	629 (17.3)	92 (14.6)	537 (85.4)	Reference		Reference	
Perceived severity							
My child will be very sick if they get HFMD							
Yes	1,842 (50.8)	632 (34.3)	1,210 (65.7)	1.72 (1.49–1.99)	<0.001	1.45 (1.19–1.76)	<0.001
No	1,784 (49.2)	415 (23.3)	1,369 (76.7)	Reference		Reference	
HFMD infection can affect my child’s schooling							
Yes	3,261 (89.9)	983 (30.1)	2,278 (69.9)	2.03 (1.53–2.69)	<0.001	1.07 (0.76–1.51)	0.700
No	365 (10.1)	64 (17.5)	301 (82.5)	Reference		Reference	
Perceived benefits							
HFMD can be prevented							
Yes	3,385 (93.4)	1,022 (30.2)	2,363 (69.8)	3.74 (2.45–5.69)	<0.001	1.01 (0.56–1.82)	0.975
No	241 (6.6)	25 (10.4)	216 (89.6)	Reference		Reference	
EV-71 Vaccination is effective in preventing HFMD							
Yes	3,313 (91.4)	1,025 (30.9)	2,288 (69.1)	5.93 (3.82–9.20)	<0.001	2.73 (1.52–4.90)	<0.001
No	313 (8.6)	22 (7.0)	291 (93.0)	Reference		Reference	
Perceived barriers							
I do not have time to take my child to get the EV-71 vaccine							
Yes	986 (27.2)	203 (20.6)	783 (79.4)	Reference	<0.001	Reference	
No	2,640 (72.8)	844 (32.0)	1,796 (68.0)	1.81 (1.52–2.16)		1.39 (1.12–1.71)	0.002
Worry about the side effects							
Yes	1,985 (54.7)	396 (19.9)	1,589 (80.1)	Reference	<0.001	Reference	
No	1,641 (45.3)	651 (39.7)	990 (60.3)	2.64 (2.28–3.06)		1.56 (1.23–1.97)	<0.001
Concern about the safety of the EV-71 vaccine							
Yes	1,920 (53.0)	367 (19.1)	1,553 (80.9)	Reference	<0.001	Reference	
No	1,706 (47.0)	680 (39.9)	1,026 (60.1)	2.81 (2.42–3.26)		1.65 (1.30–2.08)	<0.001
EV-71 vaccine is expensive							
Yes	1,960 (54.1)	324 (16.5)	1,636 (83.5)	Reference	<0.001	Reference	
No	1,666 (45.9)	723 (43.4)	943 (56.6)	3.87 (3.32–4.51)		2.94 (2.45–3.53)	<0.001
Cues to action							
I often get information from health education program about EV-71 vaccination							
Yes	2,246 (61.9)	757 (33.7)	1,489 (66.3)	1.91 (1.63–2.23)	<0.001	1.21 (0.99–1.50)	0.069
No	1,380 (38.1)	290 (21.0)	1,090 (79.0)	Reference		Reference	
Medical staff recommended that I shall get my child vaccinated with EV-71 vaccine							
Yes	2,690 (74.2)	887 (33.0)	1,803 (67.0)	2.39 (1.98–2.88)	<0.001	1.77 (1.40–2.24)	<0.001
No	936 (25.8)	160 (17.1)	776 (82.9)	Reference		Reference	

† Hosmer–Lemeshow test, chi-square: 6.011, value of *p*: 0.646; Nagelkerke *R*^2^: 0.287.

Most of the constructs in the HBM were significantly associated with having a definite intention to vaccinate a child with the EV-71 vaccine in the multivariable analysis. Disagreeing on the high cost of the vaccine (OR = 2.94, 95% CI 2.45–3.53) under the perceived barriers construct was the strongest predictor for a definite intention. Having a definite intention for EV-71 vaccination was significantly strongly correlated with the perception that EV-71 vaccination is effective in preventing HFMD under the perceived benefit construct (OR = 2.73, 95% CI 1.52–4.90) and worry about a child getting HFMD (OR = 1.79, 95% CI 1.34–2.370). While the cue to action was a significant construct, participants who reported receiving a recommendation from medical staff (OR = 1.77, 95% CI 1.40–2.24) were associated with having a more definite intention to vaccinate.

### 3.5 Willingness-to-pay (WTP)

**Figure 3:**
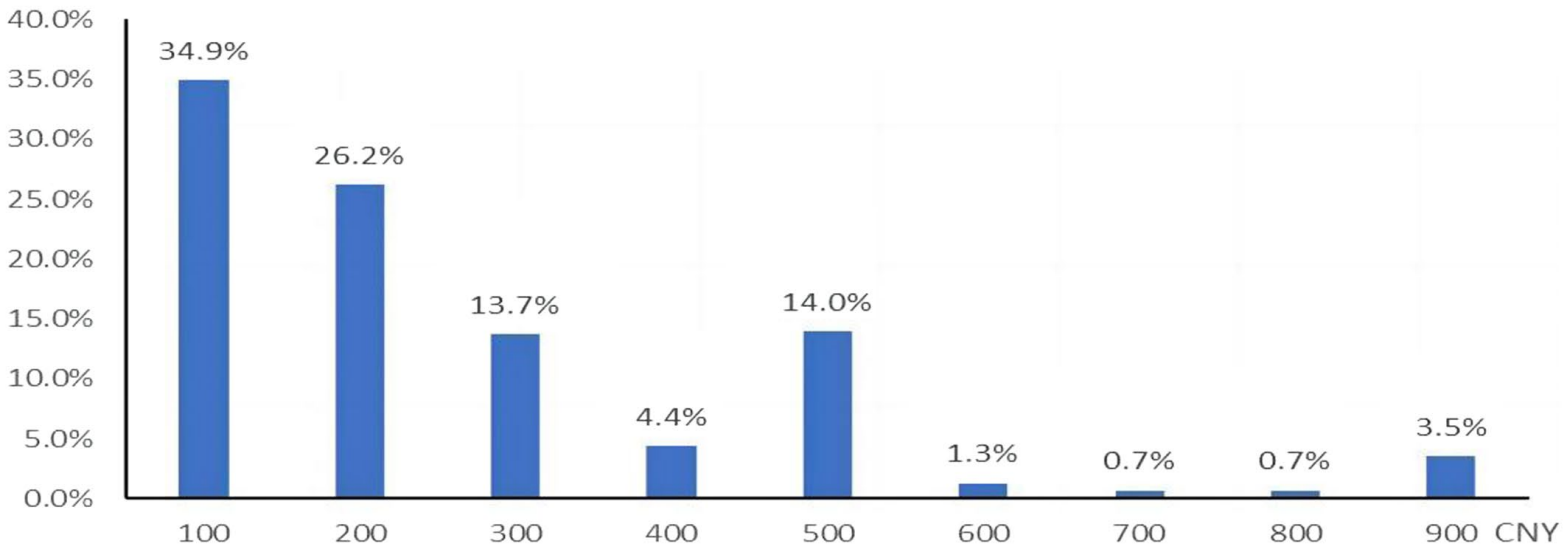
Willingness-to-pay for the EV-71 vaccination (*N* = 3,626).

Figure 3 shows that most of the participants were willing to pay an amount of either CNY¥100 [USD$14] (34.9%) or CNY¥ 200 [USD$ 28] (26.2%) for an EV-71 vaccine. The median WTP for the EV-71 vaccine was CNY¥ 200/USD$ 28 (IQR CNY¥ 100-400/USD$ 14-56). Table 3 shows the results of the univariable and multivariable regression analyses for the marginal WTP an amount of CNY¥ 100/200 [USD$ 14/28], CNY¥ 300/400 [USD$ 42/56], and CNY¥ 500 [USD$ 70] by demographics and HBM constructs. The results of the multinomial logistic regression revealed that participants with households registered in other provinces had a higher WTP an amount of CNY¥ 300/400 [USD$ 42/56] and CNY¥ 500 [USD$ 70] over CNY¥ 100/200 [USD$ 14/28]. The participants with an annual household income level of ≥CNY¥ 16,000 had the highest WTP for an amount of CNY ¥500 [USD$72] over CNY¥ 100/200 [USD$14/28] (OR = 3.23, 95%CI: 2.02–5.15). Other demographic factors positively associated with WTP an amount of CNY¥ 500 [USD$ 72] over CNY¥ 100/200[USD$ 14/28] were higher education level (OR = 1.39, 95% CI 1.09–1.76), only having one child aged 0–5 years in the family (OR = 1.45, 95% CI 1.12–1.88) and having a child aged less than 3 years (OR = 1.66, 95% CI 1.29–2.31).

**Table 3:** Multinomial logic regression of factors associated with marginal willingness-to-pay (WTP) for EV-71 vaccine (*N* = 3,626).

	Univariate analysis				Multivariate logistic regression^†^			
	Marginal WTP				Marginal WTP			
	CNY¥100/200 (*N* = 2,221)	CNY¥300/400 (*N* = 662)	≥CNY¥500 (*N* = 743)	*p*-value	CNY¥300/400 OR (95% CI)	*p*-value	≥CNY¥500 OR (95% CI)	*p*-value
Social demographic characteristics								
Guardian factor								
Age (years)								
<30	575 (54.8)	207 (19.7)	267 (25.5)	<0.001	1.17 (0.79–1.73)	0.432	1.57 (1.00–2.47)	0.049
30–40	1,343 (63.0)	370 (17.3)	420 (19.7)		0.97 (0.68–1.39)	0.862	1.12 (0.74–1.72)	0.587
>40	303 (68.2)	85 (19.1)	56 (12.6)		Reference		Reference	
Relationship								
Father	505 (57.7)	162 (18.5)	208 (23.8)	<0.001	0.74 (0.44–1.25)	0.262	0.97 (0.49–1.90)	0.919
Mother	1,589 (62.0)	459 (17.9)	515 (20.1)		0.67 (0.40–1.11)	0.122	0.71 (0.36–1.39)	0.317
Other guardians	127 (67.6)	41 (21.8)	20 (10.6)		Reference		Reference	
Birthplace								
Urban	674 (55.8)	238 (19.7)	296 (24.5)	<0.001	1.06 (0.86–1.31)	0.595	1.05 (0.84–1.31)	0.687
Rural	1,547 (64.0)	424 (17.5)	447 (18.5)		Reference		Reference	
Workplace								
Urban	1,252 (56.0)	433 (19.4)	551 (24.6)	<0.001	1.14 (0.91–1.43)	0.250	1.19 (0.93–1.53)	0.168
Rural	969 (69.7)	229 (16.5)	192 (13.8)		Reference		Reference	
Census register place								
Fujian Province	2,086 (62.1)	603 (17.9)	672 (20.0)	0.002	Reference		Reference	
Other provinces	135 (50.9)	59 (22.3)	71 (26.8)		1.46 (1.03–2.05)	0.033	1.52 (1.05–2.19)	0.025
Occupation								
Professionals and managerial	514 (56.4)	179 (19.6)	218 (23.9)	<0.001	1.04 (0.75–1.43)	0.820	1.08 (0.76–1.53)	0.676
Clerks	114 (48.1)	52 (21.9)	71 (30.0)		1.11 (0.72–1.72)	0.631	1.09 (0.70–1.53)	0.695
Industrial workers	213 (67.0)	48 (15.1)	57 (17.9)		0.74 (0.49–1.11)	0.142	1.09 (0.70–1.72)	0.604
Self-employed	236 (57.4)	73 (17.8)	102 (24.8)		0.87 (0.61–1.26)	0.465	1.13 (0.72–1.75)	0.482
Farmers	319 (74.0)	72 (16.7)	40 (9.3)		1.00 (0.69–1.45)	0.994	1.15 (0.78–1.70)	0.550
Service personnel	100 (61.0)	28 (17.1)	36 (22.0)		0.86 (0.53–1.42)	0.564	1.13 (0.67–1.90)	0.650
Others occupation	279 (56.8)	91 (18.5)	121 (24.6)		1.08 (0.77–1.50)	0.666	1.42 (0.99–2.04)	0.058
Housewife/ retiree/ unemployed	446 (67.3)	119 (17.9)	98 (14.8)		Reference		Reference	
Highest education level								
High school and below	1,242 (67.3)	327 (17.7)	276 (15.0)	<0.001	Reference		Reference	
University and above	979 (55.0)	335 (18.8)	467 (26.2)		1.01 (0.80–1.28)	0.954	1.39 (1.09–1.76)	0.010
Average annual household income (CNY¥)								
<4,000	829 (77.4)	144 (13.4)	98 (9.2)	<0.001	Reference		Reference	
4,000–7,999	853 (59.4)	292 (20.3)	291 (20.3)		0.78 (0.48–1.27)	0.573	1.03 (0.68–1.57)	0.873
8,000–15,999	465 (48.8)	198 (16.9)	290 (30.4)		0.87 (0.54–1.40)	0.326	1.41 (0.93–2.14)	0.110
≥16,000	74 (44.6)	28 (16.9)	64 (38.6)		1.60 (0.96–2.66)	0.065	3.23 (2.02–5.15)	<0.001
Number of children aged 0–5 in the family								
1	1,759 (60.3)	537 (18.4)	620 (21.3)	0.034	1.15 (0.91–1.45)	0.254	1.45 (1.12–1.88)	0.004
>1	462 (65.1)	125 (17.6)	123 (17.3)		Reference		Reference	
Knowledge score of HFMD								
High (12–14)	1,060 (60.1)	338 (19.2)	367 (20.8)	0.293				
Low (0–11)	1,161 (62.4)	324 (17.4)	376 (20.2)					
Children’s factor								
Child’s gender								
Boy	1,249 (60.7)	389 (18.9)	419 (20.4)	0.504				
Girl	972 (62.0)	273 (17.4)	324 (20.7)					
Child’s age (years)								
<3	1,182 (56.6)	397 (19.0)	510 (24.4)	<0.001	1.09 (0.87–1.37)	0.474	1.66 (1.29–2.13)	<0.001
3–5	1,039 (67.6)	265 (17.2)	233 (15.2)		Reference		Reference	
Child’s physical condition								
Healthy	2,045 (61.1)	613 (18.3)	690 (20.6)	0.749				
Frequently sick	176 (63.3)	49 (17.6)	53 (19.1)					
Child’s category								
Nursery children	1,088 (62.5)	304 (17.5)	349 (20.0)	0.312				
Non-nursery children	1,133 (60.1)	358 (19.0)	394 (20.9)					
Child’s HFMD history								
Yes	216 (68.8)	50 (15.9)	48 (15.3)	0.011	Reference		Reference	
No	2,005 (60.5)	612 (18.5)	695 (21.0)		1.25 (0.88–1.77)	0.217	1.16 (0.79–1.71)	0.458
Other children around who have suffered from HFMD								
Yes	712 (62.8)	199 (17.6)	222 (19.6)	0.414				
No/unsure	1,509 (60.5)	463 (18.6)	521 (20.9)					
Health belief								
Perceived susceptibility								
My child has a high chance of getting HFMD								
Yes	985 (59.8)	326 (19.8)	337 (20.4)	0.085				
No	1,236 (62.5)	336 (17.0)	406 (20.5)					
Worry about my child getting HFMD								
Yes	1,781 (59.4)	558 (18.6)	658 (22.0)	<0.001	1.37 (1.03–1.81)	0.032	1.82 (1.33–2.49)	<0.001
No	440 (70.0)	104 (16.5)	85 (13.5)		Reference		Reference	
Perceived severity								
My child will be very sick if they get HFMD								
Yes	1,092 (59.3)	358 (19.4)	392 (21.3)	0.042	1.16 (0.93–1.45)	0.181	1.06 (0.84–1.34)	0.607
No	1,129 (63.3)	304 (17.0)	351 (19.7)		Reference		Reference	
HFMD infection can affect my child’s schooling								
Yes	1,987 (60.9)	583 (17.9)	691 (21.2)	0.003	0.79 (0.56–1.10)	0.167	1.16 (0.77–1.75)	0.478
No	234 (64.1)	79 (21.6)	52 (14.2)		Reference		Reference	
Perceived benefits								
HFMD can be prevented								
Yes	2,058 (60.8)	606 (17.9)	721 (21.3)	<0.001	0.65 (0.41–1.03)	0.068	1.12 (0.59–2.12)	0.738
No	163 (67.6)	56 (23.2)	22 (9.1)		Reference		Reference	
EV-71 vaccination is effective in preventing HFMD								
Yes	1,996 (60.2)	600 (18.1)	717 (21.6)	<0.001	1.15 (0.75–1.79)	0.521	2.09 (1.16–3.76)	0.014
No	225 (71.9)	62 (19.8)	26 (8.3)		Reference		Reference	
Perceived barriers								
I do not have time to take my child to get EV-71 vaccine								
Yes	678 (68.8)	176 (17.8)	132 (13.4)	<0.001	Reference		Reference	
No	1,543 (58.4)	486 (18.4)	611 (23.1)		1.05 (0.84–1.31)	0.687	1.26 (0.98–1.63)	0.073
Worry about the side effects								
Yes	1,331 (67.1)	343 (17.3)	311 (15.7)	<0.001	Reference		Reference	
No	890 (54.2)	319 (19.4)	432 (26.3)		0.90 (0.69–1.17)	0.422	1.07 (0.81–1.41)	0.653
Concern about the safety of the EV-71 vaccine								
Yes	1,313 (68.4)	316 (16.5)	291 (15.2)	<0.001	Reference		Reference	
No	908 (53.2)	346 (20.3)	452 (26.5)		1.25 (0.97–1.63)	0.090	1.09 (0.82–1.44)	0.560
EV-71 vaccine is expensive								
Yes	1,550 (79.1)	299 (15.3)	111 (5.7)	<0.001	Reference		Reference	
No	671 (40.3)	363 (21.8)	632 (37.9)		2.58 (2.11–3.17)	<0.001	10.86 (8.49–13.88)	<0.001
Cues to action								
I often get information from health education program about EV-71 vaccination								
Yes	1,366 (60.8)	409 (18.2)	471 (21.0)	0.653				
No	855 (62.0)	253 (18.3)	272 (19.7)					
Medical staff recommended that I shall get my child vaccinated with EV-71 vaccine								
Yes	1,627 (60.5)	509 (18.9)	554 (20.6)	0.162				
No	594 (63.5)	153 (16.3)	189 (20.2)					

† Multinomial regression; reference group: CNY¥100/200.

Goodness of fit; Pearson’s chi-square: 7386.496, *p*-value: 0.002; Nagelkerke *R*^2^: 0.300.

For HBM constructs, a higher marginal WTP was significantly associated with the perception of susceptibility, benefits, and barriers. A disagreement that the EV-71 vaccine is expensive was the strongest facilitator that was associated with higher WTP for an amount of CNY¥ 300/400 [USD$ 42/56] (OR = 2.58, 95%CI: 2.11–3.17) and CNY¥ 500 [USD$ 70] (OR = 10.86, 95%CI: 8.49–13.88) over CNY¥ 100/200 [USD$ 14/28]. Concern about a child getting HFMD under the construct of perceived susceptibility was another factor significantly associated with a higher WTP for an amount of CNY¥ 300/400 [USD$ 42/56] (OR = 1.37, 95%CI: 1.03–1.81) and CNY¥ 500 [US$ 70] (OR = 1.82, 95%CI: 1.33–2.49). Participants who considered the EV-71 vaccine to be effective in preventing HFMD had a higher chance to pay for CNY¥ 500 [US$ 70] and above (OR = 2.09, 95%CI: 1.16–3.76) over CNY¥ 100/200 [US$ 14/28].

## 4 Discussion

Parental perspectives and beliefs about vaccines are important factors in predicting a child’s immunization status. The current study explored the knowledge level of HFMD and the EV-71 vaccine, as well as their intent and WTP to vaccinate their children aged 0–5 years with the EV-71 vaccine, among parents/guardians in southeastern China.

The majority (87.7%) of participants were intent to vaccinate their children with the EV-71 vaccine, which is higher than the observed vaccination rates of 4.2 and 8.5% in Fujian Province in 2017 and 2018, respectively (17). Among those who were not willing to vaccinate their children, concern about vaccine safety was the major reason. In 2016, an illegal vaccine case was reported in China. Vaccines were sold in many provinces and cities without precise cold chain storage and transportation, including a variety of class II vaccines for children. Such news has widely caused parental concerns about the safety of vaccines used for their children (23, 33). The results of our study also showed that concern about the safety of the EV-71 vaccine was significantly correlated with parental intent to vaccinate their children, which is in line with the findings reported by Li et al. (31). Furthermore, the perception that the EV-71 vaccine can effectively prevent HFMD and worry about children suffering from HFMD are the most critical factors affecting the intention to vaccinate. The results of our study have proved that healthcare organizations are the most important source of information on the EV-71 vaccine (58.7%), and recommendations from healthcare professionals can significantly increase parental intention to vaccinate their children with the EV-71 vaccine (OR = 1.74, 95%CI: 1.46–2.32). These findings are consistent with other research on vaccine acceptability (34, 35). In addition, willingness to vaccinate was higher among those with high knowledge scores of HFMD (OR = 1.90, 95%CI: 1.57–2.29) in our current study. Therefore, health education programs in healthcare institutions such as community health centers, where medical staff could provide easy-to-understand education to parents, may be necessary to increase the uptake rate of the EV-71 vaccine. Meanwhile, the government could organize national campaigns to spread the message that “vaccines are good,” which could also help to eliminate vaccine hesitancy among the public.

The results of this study showed that parents with higher education levels had a higher acceptance rate of the EV-71 vaccine. Education increases knowledge and awareness of vaccine benefits; people with a high education level were more likely to receive information about the EV-71 infection and the HFMD vaccine from different sources (36). However, some studies agree that high levels of education are positively correlated with vaccine hesitancy (37, 38). Other studies found no statistically significant differences between high and low levels of education (39). Many vaccine-preventable diseases have almost been eradicated in countries with high economic development and educational levels, which might decrease the perceived threat of communicable diseases among the public and increase vaccine skepticism (40). On the other hand, the number of reported cases and deaths related to HFMD ranked first among the category C infectious diseases in China, causing serious economic and social burdens (41). HFMD is still an important threat to public health in China and many other developing countries. In fact, previous studies have shown that the majority of the EV-71 vaccine-associated adverse events reported either in China or globally were mild or self-limiting and that the EV-71 vaccine has been proven to have good safety (16, 42). Therefore, healthcare professionals could emphasize the safety aspect of the EV-71 vaccine to parents to correct parental misconceptions, which might be valuable to enhance their confidence in vaccinating their children.

The affordability of the vaccine is also an important factor influencing parents’ willingness to vaccinate their children with the EV-71 vaccine. In the current study, approximately 80% of respondents were willing to pay CNY¥ 100–400 [USD$14–56], and only 20% of them were willing to pay CNY¥ 500 [USD$70] and above. The estimated mean WTP for the EV-71 vaccine was CNY¥ 268 [USD$ 38], which is lower than that in Malaysia [USD$ 87.47] (43). However, it is worth mentioning that, as approximately 70% of our study participants had household income lower than CNY¥ 8000 [USD$ 1143], a vaccine cost of CNY¥ 268 [USD$ 38] could be their whole-day family income and, therefore, could still be too expensive for those families with low household income. In China, the EV-71 vaccine is a class II vaccine that must be purchased by parents at their own expense. The cost is approximately CNY¥ 500 [US$ 70] for a child to complete two doses of vaccination, which is higher than the price that most parents/guardians in this study were willing to pay. The current high cost of EV-71 vaccine appears to be a significant barrier for parents to vaccinate their children, suggesting that children in China can be widely vaccinated with the EV-71 vaccine when the price stands at USD$ 25 or less (44). Within the price range of current routine vaccines paid by the Chinese government, a national EV-71 vaccination program would be cost-saving or highly cost-effective in reducing the EV-71 vaccine-related morbidity, mortality, and use of health services among children younger than 5 years in China (45). Policymakers may consider the EV-71 vaccination as part of a routine childhood immunization schedule.

Several study limitations should be noted. First, this study was a cross-sectional study, and causality could not be determined. Second, the questionnaire in this study was self-administered, and respondents may have chosen options that were socially expected but deviated from their true beliefs. Third, parents’ intent to vaccinate their children (87.7%) was higher than the actual vaccination rates of 4.2 and 8.5% that were observed in Fujian Province in 2017 and 2018, respectively. This gap may be related to parents’ participation in this study, influencing their understanding of EV-71 disease and its vaccine. Fourth, the generalization of study results may be limited as our current study only recruited participants from one province in southeastern China. Further study with a nationwide study design could be valuable. The strengths of this study are also worth mentioning. First, we used stratified and systematic sampling methods to recruit study participants, which helped to ensure the representativeness of study participants. Second, the questionnaire has shown a high internal consistency (Cronbach’s alpha of 0.915). Third, standard training was provided to all medical staff who were recruited to conduct data collection.

## 5 Conclusion

Health promotion is warranted to promote the EV-71 vaccination; among parents/guardians who expressed the intention to vaccinate their children, two-thirds expressed probable intention. The HBM can be used to develop strategies for enhancing EV-71 vaccine uptake and WTP. Most importantly, the findings revealed that interventions targeting perceived barriers, i.e., the efficacy and affordability of the EV-71 vaccine, are most crucial. Concerns regarding the side effects or safety of vaccines were also significant predictors of EV-71 vaccination intent, implying that the government will put effort into enhancing public trust. The WTP was positively related to socioeconomic factors, which should guide policy recommendations for the future national EV-71 improvisation program in China. Further follow-up studies need to be conducted to determine parents’ actual vaccination rate of the EV-71 vaccine for their children.

## Data availability statement

The raw data supporting the conclusions of this article will be made available by the authors, without undue reservation.

## Ethics statement

The studies involving humans were approved by the Ethics Committee of Fujian Provincial Center for Disease Control and Prevention. The studies were conducted in accordance with the local legislation and institutional requirements. The participants provided their written informed consent to participate in this study.

## Author contributions

LC: Conceptualization, Data curation, Formal analysis, Investigation, Methodology, Resources, Software, Writing – original draft, Writing – review & editing. SZ: Data curation, Formal analysis, Investigation, Methodology, Validation, Visualization, Writing – review & editing. XX: Conceptualization, Data curation, Formal analysis, Investigation, Methodology, Resources, Software, Writing – original draft, Writing – review & editing. JL: Conceptualization, Data curation, Formal analysis, Investigation, Writing – original draft, Writing – review & editing. FX: Conceptualization, Project administration, Supervision, Writing – original draft, Writing – review & editing. YL: Conceptualization, Data curation, Formal analysis, Investigation, Methodology, Resources, Software, Supervision, Validation, Visualization, Writing – original draft, Writing – review & editing. DZ: Conceptualization, Data curation, Formal analysis, Funding acquisition, Investigation, Methodology, Resources, Software, Supervision, Validation, Visualization, Writing – original draft, Writing – review & editing.
